# Gut Microbial Signatures for Glycemic Responses of GLP-1 Receptor Agonists in Type 2 Diabetic Patients: A Pilot Study

**DOI:** 10.3389/fendo.2021.814770

**Published:** 2022-01-10

**Authors:** Chih-Yiu Tsai, Hsiu-Chen Lu, Yu-Hsien Chou, Po-Yu Liu, Hsin-Yun Chen, Meng-Chuan Huang, Chia-Hung Lin, Chi-Neu Tsai

**Affiliations:** ^1^ Division of Endocrinology and Metabolism, Chang Gung Memorial Hospital, Taoyuan, Taiwan; ^2^ Graduate Institute of Clinical Medical Sciences, Chang Gung University, Taoyuan, Taiwan; ^3^ Department of Life Science, College of Life Science, National Taiwan University, Taipei, Taiwan; ^4^ Department of Internal Medicine, National Taiwan University College of Medicine, Taipei, Taiwan; ^5^ Department of Pathobiology and Population Sciences, Royal Veterinary College, University of London, Hatfield, United Kingdom; ^6^ Department of Medical Nutrition Therapy, Chang Gung Memorial Hospital, Taoyuan, Taiwan; ^7^ Department of Nutrition and Dietetics, Kaohsiung Medical University Hospital, Kaohsiung, Taiwan; ^8^ Graduate Institute of Medicine and Department of Public Health and Environmental Medicine, School of Medicine, Kaohsiung Medical University, Kaohsiung, Taiwan; ^9^ Department of Chinese Medicine, College of Medicine, Chang Gung University, Taoyuan, Taiwan; ^10^ Department of Surgery, New Taipei Municipal Tucheng Hospital, New Taipei City, Taiwan

**Keywords:** gut microbiota, GLP-1 receptor agonists, type 2 diabetes mellitus, glycemic response, dysbiosis, GLP-1 resistance

## Abstract

**Backgrounds:**

Glucagon-like peptide-1 receptor agonist (GLP-1 RA) is probably one of more effective antidiabetic agents in treatment of type 2 diabetes mellitus (T2D). However, the heterogenicity in responses to GLP-1 RA may be potentially related to gut microbiota, although no human evidence has been published. This pilot study aims to identify microbial signatures associated with glycemic responses to GLP-1 RA.

**Materials and Methods:**

Microbial compositions of 52 patients with T2D receiving GLP-1 RA were determined by 16S rRNA amplicon sequencing. Bacterial biodiversity was compared between responders versus non-responders. Pearson’s correlation and random forest tree algorithm were used to identify microbial features of glycemic responses in T2D patients and multivariable linear regression models were used to validate clinical relevance.

**Results:**

Beta diversity significantly differed between GLP-1 RA responders (*n* = 34) and non-responders (*n* = 18) (ADONIS, *P* = 0.004). The top 17 features associated with glycohemoglobin reduction had a 0.96 diagnostic ability, based on area under the ROC curve: *Bacteroides dorei* and *Roseburia inulinivorans*, the two microbes having immunomodulation effects, along with *Lachnoclostridium* sp. and *Butyricicoccus* sp., were positively correlated with glycemic reduction; *Prevotella copri*, the microbe related to insulin resistance, together with *Ruminococcaceae* sp., Bacteroidales sp., *Eubacterium coprostanoligenes* sp., *Dialister succinatiphilus*, *Alistipes obesi*, *Mitsuokella* spp., *Butyricimonas virosa*, *Moryella* sp., and *Lactobacillus mucosae* had negative correlation. Furthermore, *Bacteroides dorei*, *Lachnoclostridium* sp. and *Mitsuokella multacida* were significant after adjusting for baseline glycohemoglobin and C-peptide concentrations, two clinical confounders.

**Conclusions:**

Unique gut microbial signatures are associated with glycemic responses to GLP-RA treatment and reflect degrees of dysbiosis in T2D patients.

## Introduction

An increasing incidence of type 2 diabetes mellitus (T2D) is a severe health issue worldwide, causing high morbidity and mortality with resulting healthcare costs expected to reach US$825B annually by 2030 (1). Therefore, glycemic control in T2D patients is critical to reduce diabetic complications, cardiovascular consequences, and costs (2).

Glucagon-like peptide-1 receptor agonist (GLP-1 RA) has pleotropic effects on pancreas, brain, and other target organs, acting through systemic or enteric neuroendocrine cell pathways (3). Augmented GLP-1 actions include anti-inflammatory, potent glycemic and body weight reduction effects (4); therefore, GLP-1 RA is highly recommended for T2D patients with cardiovascular risk factors (5). However, responses to GLP-1 RA are heterogeneous, with ~ 30 to 50% of patients having an inadequate response or treatment failure (6, 7), representing both a treatment barrier and an economic burden (8). Perhaps loss of efficacy is due to target cells becoming resistant to GLP-1 (9). Risk factors for development of GLP-1 RA treatment failure are prolonged disease duration, previous insulin use, lower C-peptide concentrations or positive islet autoantibodies, partially implicating β-cell failure (10, 11). Regardless, apart from insulin deficiency, GLP-1 resistance has no specific marker.

Gut microbiota is increasingly implicated in the pathogenesis of T2D. Based on metagenome-wide association studies, there were decreased abundances of some butyrate-producing bacteria and increased opportunistic pathogens in patients with T2D (12). Gut microbial dysbiosis could impair gut barriers and cause endotoxemia, insulin resistance and hyperglycemia (13). For T2D patients with different degrees of severity, gut microbiota could serve as an indicator. Furthermore, there is an interplay between gut microbiota and some antidiabetic agents. Metformin is the first example showing that T2D patients with different microbiota may have diverse treatment efficacy (14). As a novel biomarker, gut microbiota may be implicated in responses to GLP-1 RA treatment as well, but there is no human evidence.

Interactions between gut microbiota and GLP-1 RA have been investigated in few animal models (15, 16). In a study of diabetic mice, microbiota-induced vagal afferent neural impairment decreased incretin effects, implying gut dysbiosis may cause GLP-1 resistance *via* a gut-brain axis (17). As GLP-1 resistance is relatively common and gut microbiota composition varies widely among populations (18), perhaps specific gut microbial signatures determine responses to GLP-1 RA treatment. However, there are insufficient data to access relationships between gut microbiota composition and GLP-1 RA treatment efficacy (19, 20). Therefore, in the pilot study, we analyzed gut microbiota of T2D patients treated with GLP-1 RA and determined clinical implications of gut microbiota in patients with distinct responses.

## Materials and Methods

### Participants’ Enrollment and Clinical Data Collection

All participants (*n* = 52) were enrolled from outpatient departments at the Taipei, Linkou and Taoyuan branches of Chang Gung Memorial Hospital, certified Diabetes Health Promotion Centers in Taiwan. Individualized management was performed by diabetes care teams. Main inclusion criteria included diagnosis of T2D, age > 20 years and currently on GLP-1 RA (liraglutide or dulaglutide) treatments with successful adherence. Main exclusion criteria included recent gastrointestinal discomfort (e.g., abdominal pain or diarrhea) within the previous month, recent use of antibiotics, use of probiotics or prebiotics within the previous month, and advanced chronic kidney disease or other metabolic disorders (thyroid dysfunction, Cushing syndrome, acromegaly, and pheochromocytoma). An informed consent form approved by the Institutional Review Board of Chang Gung Memorial Hospital was signed by each participant (certificate number: 201900467B0).

Baseline characteristics before GLP-1 RA treatment including age, gender, duration of diabetes mellitus, body mass index (BMI), serum creatinine, alanine aminotransferase, fasting plasma glucose, glycohemoglobin (HbA1c), C-peptide, lipid profiles, urine albumin-to-creatinine ratio (UACR) and concurrent antidiabetic medication use were recorded. Kidney function, as assessed with eGFR (estimated glomerular filtration rate), was calculated with the IDMS traceable MDRD-study equation. A validated 45-item food frequency questionnaire designed for T2D patients of Han Chinese descent was used to evaluate habitual dietary contents (21). Each participant completed the questionnaire with assistance from a certified dietician. Raw data was transformed into daily dietary fiber intake and estimated daily calorie intake, apportioned among protein, fat, and carbohydrate.

Changes in HbA1c and BMI from the baseline to week 12 were reviewed. To adjust the impact of baseline HbA1c concentration on decreased blood glucose concentrations, GLP-1 RA responders were defined as an HbA1c reduction ratio [(HbA1c level at week 12) – (HbA1c level at baseline)/(HbA1c level at baseline)] ≥ 0.12, whereas GLP-1 RA non-responders were defined as an HbA1c reduction ratio < 0.12. This cut-off ratio was adopted from approximate HbA1c concentrations associated with a 1% change from an 8% HbA1c baseline (22, 23).

To have enough responders and non-responders to identify the relationships between their gut microbiota and treatment effects of GLP-1 RA, we used information from a Taiwan cohort study to estimate sample sizes (24). A sample size of 45 patients with 2:1 ratio of responders (*n* = 30) and non-responders (*n* = 15) was calculated, based on 70% power to detect superiority of responders versus non-responders on the 12% reduction HbA1c ratio from baseline (mean = 9.6 with a standard deviation of 1.6) and a one-sided α of 0.05.

### Stool Collection and DNA Extraction

A stool sample (0.5 to 3 g) was collected from each participant using a Longsee Profecal kit (Longsee Biomedical Corporation, Guangzhou, China) and processed and stored in accordance with the manufacturer’s protocol. Non-human DNA was extracted from the stool with a QIAamp^®^ DNA Stool Mini Kit (Qiagen, Hilden, Germany), according to manufacturer’s recommendations. The suspension was heated (5 min at 95°C) to lyse Gram positive bacteria, followed by a beads-beating process with Roche MagNALyser (6500 rpm, 30 seconds, 3 times, with 60 seconds cooling on ice). Final DNA products were stored at −20°C.

### 16S rRNA Gene Amplicon Sequencing

Library preparation for 16s rRNA gene V3 and V4 regions amplicon sequencing was done according to the Illumina protocol (25). Microbial genomic DNA samples (8 to 15 ng) were prepared. A two-step polymerase chain reaction (PCR) workflow of amplicon PCR, first PCR clean-up, index PCR, second PCR clean-up and library validation, was done. Pooled libraries were sequenced on the Illumina Miseq platform (Illumina, San Diego, CA, USA) with v3 reagents for paired-end sequencing (2 × 300 bps). All 16S library preparation and sequencing were done and validated by the Genomic Medicine Core Laboratory, Chang Gung Memorial Hospital at Linkou.

### Processing Sequence Reads

Raw sequence reads were acquired from libraries and processed with QIIME 2 version 2020.2, following the Amplicon SOP v2 of microbiome helper (26, 27). Raw paired-end demultiplexed reads were first trimmed using Cutadapt QIIME 2 plugin to remove primer sequences from reads and from those that did not begin with a primer sequence (28), followed by denoising into amplicon sequence variants (ASVs) using DADA2 to exclude low-quality reads. Taxonomy was assigned to the representative ASV sequences using the Naïve Bayesian classifier against the SILVA database (v. 132) (29, 30). In total, 5,503,725 high quality paired-end sequence reads were obtained after filtering out rare, contaminant and unclassified ASVs, ranging from 48,155 to 205,487 per sample. Then, the filtered table was rarefied to minimum sample-length of reads per sample. After QIIME 2 pipeline processing, 1,553 ASVs shared among 52 subjects were identified for analysis.

### Microbiota and Statistical Analyses

R software and packages were used for subsequent microbiota analyses on the final filtered table obtained from the QIIME 2 pipeline (31). The R package *vegan* was used to calculate alpha and beta diversities (32). Observed ASV numbers and Shannon index were used to calculate alpha diversity and a Wilcoxon rank sum test was done to detect differences (α = 0.05) between responders and non-responders. Principal coordinate analysis (PCoA) with Bray-Curtis dissimilarity of each sample was performed to compare, between the two groups, beta diversity of gut microbiota; these were compared using an ADONIS (permutational multivariate analysis of variance using distance matrices) method (33). The Firmicutes/Bacteroidetes ratio and relative abundances of bacterial genera known for association with T2D (*Bacteroides, Faecalibacterium, Roseburia, Bifidobacterium*, and *Akkermansia*) were also compared between the two groups (34, 35).

Pearson’s correlation between the HbA1c reduction ratio and the log-transformed abundance of each ASV was calculated. A prevalence of 10% was applied to exclude rare ASVs which were either with positive correlation, but present in < 10% of responders, or with negative correlation, but present in < 10% of non-responders. A random forest algorithm was further applied to filtered ASVs to select the most important features that distinguished responders versus non-responders. Receiver-operating characteristic (ROC) analysis was used to evaluate the performance of selected features and significant clinical variables as classifiers. The dataset was divided into training and validation sets in an 80:20 ratio.

The Basic Local Alignment Search Tool (BLAST) was used to verify the scientific names of ASVs selected as most important features by searching against NCBI non-redundant (nr) and rRNA databases with parameters: query coverage = 100%, percent identity > 99% and E value = 0 (access date: Jan 23, 2021) (36). R package *igraph* and *SpiecEasi* were used for construction of the co-occurrence network with SparCC correlation coefficients (37–39). Correlation coefficients were calculated by bootstrapping 99 repetitions with a defined significance (α = 0.05). Edges with coefficients > |0.2| were plotted. The R codes for plots of alpha and beta diversity, random forest, ROC analysis and network were modified from the MARco website (40).

The mean and standard error of the mean of continuous clinical variables were calculated. Wilcoxon rank sum test was applied for assessing differences (α = 0.05) between the two groups. Wilcoxon singed rank test was used to test the significant difference of paired samples, Pearson’s correlation was used to determine linear relationships between two variables and two-tailed Fisher’s Exact Test was used for testing significant differences (α = 0.05) in categorical variables between two groups. Multivariable linear regression analysis of HbA1c reduction was done. Selected microbial features and important clinical variables (*P* < 0.2) were used for model building. All variables were checked with diagnostic tests to avoid multicollinearity. Backward variable elimination was used to obtain explanatory variables in regression models.

## Results

### Clinical Characteristics and Treatment Results

**Figure 1:**
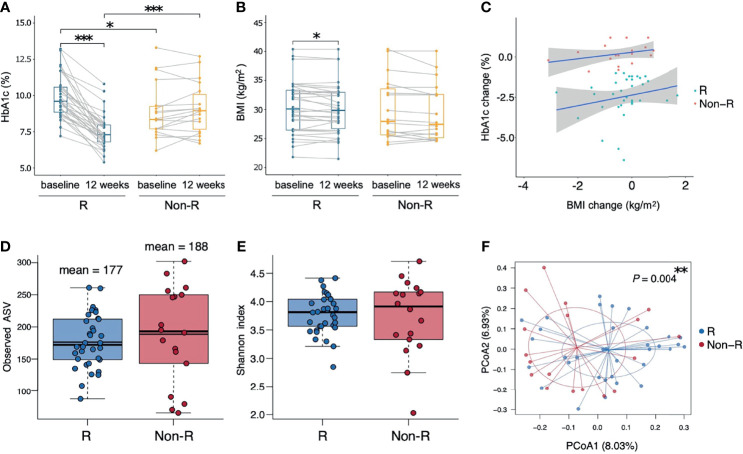
Therapeutic outcomes and microbiota biodiversity of type 2 diabetic patients receiving GLP-1 RA treatment. **(A, B)** Responders had significant reductions in both HbA1c and BMI (*P* < 0.001 and *P* = 0.041 respectively, Wilcoxon signed rank tests), whereas non-responders had insignificant changes. Responders had a higher HbA1c than non-responders at baseline and a lower HbA1c at week 12 (*P* = 0.012 and *P* < 0.001 respectively, Wilcoxon rank sum tests). **(C)** Correlations between the change from the baseline of HbA1c and BMI in the two groups were not significant (*P* > 0.05, Pearson’s correlation). **(D, E)** Similar alpha diversity of the bacterial microbiota in the two groups, based on observed ASV numbers and Shannon index (*P* = 0.376 and *P* = 0.832 respectively, Wilcoxon rank sum tests). The mean observed ASVs are shown as additional thin lines in boxes. **(F)** There was a difference in beta diversity between the two groups by PCoA (*P* = 0.004, ADONIS). Each point represented each individual, colored according to treatment responses. In all panels: *P < 0.05; **P < 0.01; ***P < 0.001. Groups are responders (R) and non-responders (Non-R). HbA1c, glycohemoglobin; BMI, body mass index; ASV, amplicon sequence variant; PCoA, principal coordinate analysis.

A total of 52 T2D patients treated with either liraglutide (*n* = 22) or dulaglutide (*n* = 30) were enrolled in this study from June, 2019 to Dec, 2020 (**Table 1**, **Supplementary Figure 1**), with 34 and 18 patients defined as responders and non-responders, respectively. Baseline HbA1c differed between groups (9.9 ± 0.2 and 8.9 ± 0.5% for responders versus non-responders, respectively; *P* = 0.012), as did triglyceride concentrations (356.6 ± 66.3 versus 145.8 ± 14 mg/ml, *P* = 0.003). Compared to baseline values, HbA1c and BMI were decreased at week 12 in responders (HbA1c: *P* < 0.001, BMI: *P* = 0.041), whereas in non-responders, there was no change in these two variables over time (HbA1c: *P* = 0.267, BMI: *P* = 0.078) (**Figures 1A, B**). Furthermore, at week 12, HbA1c differed in responders versus non-responders (7.5 ± 0.2 versus 9.1 ± 0.4%, *P* < 0.001). There were no significant correlations for change of HbA1c level from baseline and change of BMI for either responders (r = 0.06, *P* = 0.665) or non-responders (r = 0.31, *P* = 0.201; **Figure 1C**). Estimated calorie intake, macro-nutrients intake proportion, dulaglutide or liraglutide use, and concurrent medication were not different between groups (**Table 1**).

**Table 1:** Clinical characteristics of type 2 diabetic patients receiving GLP-1 RA (baseline).

	Responder (*n* = 34)	Non-responder (*n* = 18)	*P* value
Age (years)	53.8 ± 2.1	51.2 ± 2.8	0.564
Gender (male/female)	22/12	8/10	0.239
Duration of diabetes (years)	10.4 ± 1.0	11.2 ± 1.2	0.589
BMI	30.4 ± 0.8	30.0 ± 1.3	0.570
Fasting plasma glucose (mg/dL)	182.8 ± 11.1	162.7 ± 14.6	0.223
HbA1c (%)	9.9 ± 0.2	8.9 ± 0.5	0.012*
eGFR (mL/min/1.73 m²)	81.0 ± 5.9	97.5 ± 13.7	0.436
UACR	749 ± 337	663 ± 442	0.129
ALT (U/L)	39.8 ± 4.9	37.5 ± 7.8	0.496
Total cholesterol (mg/dL)	191.6 ± 11.7	160.4 ± 7.6	0.100
LDL-cholesterol (mg/dL)	98.3 ± 7.4	94.7 ± 6.5	0.953
HDL-cholesterol (mg/dL)	38.4 ± 2.0	41.6 ± 2.4	0.385
Triglyceride (mg/dL)	356.6 ± 66.3	145.8 ± 14	0.003**
C-peptide (ng/mL)	3.7 ± 0.3	2.7 ± 0.4	0.091
Estimated calorie intake (Kcal/day)	1627 ± 108	1634 ± 135	0.992
Protein (%)	17.7 ± 0.7	17.1 ± 0.8	0.615
Fat (%)	32.4 ± 1.8	31.8 ± 1.7	0.893
Carbohydrate (%)	50.0 ± 2.3	51.1 ± 2.4	0.916
Dietary fiber intake (g/day)	15.8 ± 1	15.3 ± 2	0.435
Dulaglutide/liraglutide	23/11	7/11	0.076
Concurrent medication (use/no-use):			
Metformin	30/4	17/1	0.648
Sulphonylurea	14/20	6/12	0.766
Pioglitazone	5/29	1/17	0.412
Insulin	12/22	11/7	0.088

BMI, body mass index; HbA1c, glycohemoglobin; eGFR, estimated glomerular filtration rate; UACR, urine albumin-to-creatinine ratio; ALT, alanine transaminase; LDL, low-density lipoprotein; HDL, high-density lipoprotein. Continuous data are shown as mean ± standard error of the mean. Wilcoxon rank sum test/Fisher’s Exact Test, *P < 0.05; **P < 0.01.

### Analysis of Biodiversity and Similarity of Gut Microbiota Between Groups

**Figure 2:**
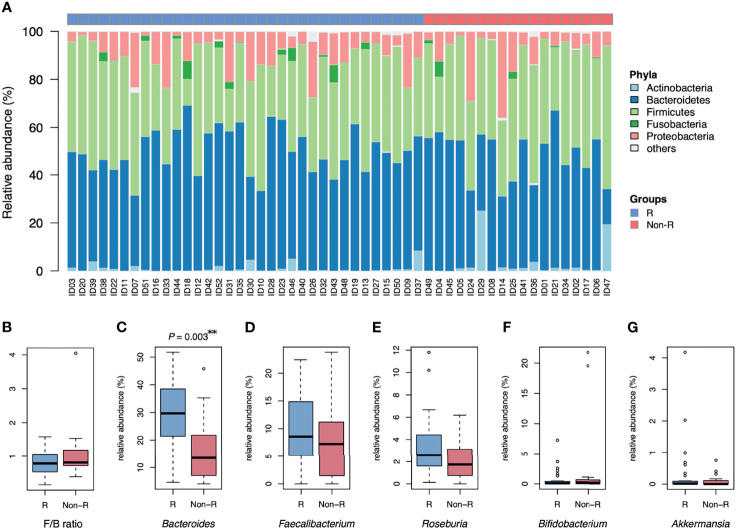
Microbiota composition of type 2 diabetic patients receiving GLP-1 RA treatment: responders and non-responders. **(A)** The barplot is relative abundance of bacteria at the phylum level. Samples are in a sequence of HbA1c reduction across the x-axis. Bacteroidetes was the dominant taxa in most samples. **(B)** The Firmicutes/Bacteroidetes ratio (F/B) was not different between groups. **(C)** The relative abundance of *Bacteroides* was higher in responders than non-responders (**P < 0.01, Wilcoxon rank sum tests). **(D, E)** No significant difference in relative abundance of *Faecalibacterium* and *Roseburia* in both groups. **(F, G)** *Bifidobacterium* and *Akkermansia* were scarce in both groups. Groups are responders (R) and non-responders (Non-R). HbA1c, glycohemoglobin.

Alpha diversity did not differ between responders versus non-responders, based on observed ASV numbers (*P* = 0.376) (**Figure 1D**) and Shannan index (*P* = 0.832) (**Figure 1E**). However, microbiota composition differed between groups (*P* = 0.004), with responders and non-responders slightly separated in the PCoA plot (**Figure 1F**). The most abundant phylum was Bacteroidetes (48.1 ± 1.5%); it was the dominant taxa at the phylum level in most samples (74% in responders and 56% in non-responders), whereas phylum Firmicutes dominated in other samples (**Figure 2A**). The Firmicutes/Bacteroidetes ratio, a hallmark of obesity, was not different between groups (responders: 0.83 ± 0.06, non-responder: 1.07 ± 0.19, *P* = 0.272; **Figure 2B**). Beta diversity was the only macro-metric marker of microbiota composition that differed between the two groups.

The relative abundance of *Bacteroides* was profoundly higher in responders than non-responders (28.5 ± 2.2 and 16.9 ± 2.8%, respectively; *P* = 0.003; **Figure 2C**). However, relative abundance of other genera known for negative association with T2D were not significantly different between groups (*Faecalibacterium*: *P* = 0.376, *Roseburia*: *P* = 0.063, *Bifidobacterium*: *P* = 0.563 and *Akkermansia*: *P* = 0.696; **Figures 2D–G**). At the bacterial genus level, *Bacteroides* was the taxa that was both dominant in abundance and had good discrimination between the two groups.

### Associations Between Gut Microbiota and Treatment Glycemic Responses

**Figure 3:**
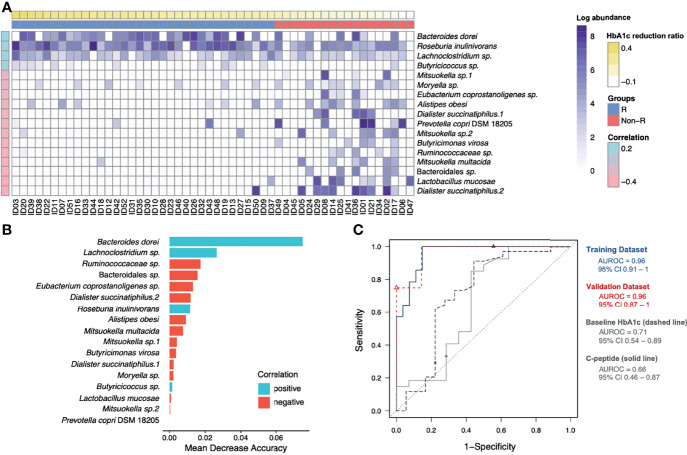
Identification of glycemic response-associated microbial signatures for GLP-1 RA treatment. **(A)** Based on the heatmap, the log-transformed abundance of 17 microbial features were significantly correlated with blood glucose reductions. Samples are in a sequence of the HbA1c reduction levels across the x-axis and 17 microbial features are in a sequence of their correlation coefficient values (*P* < 0.05, Pearson’s correlation) in the y-axis. Purple grading in a cell represents the log-transformed abundance of the microbial feature. Responders (R) and non-responders (Non-R) are colored in blue and red, respectively. **(B)** The selected 17 distinct features were plotted by their mean decrease accuracy scores, based on random forest model. The higher the value of mean decrease accuracy score was, the higher importance of the microbial features in the model ranked. **(C)** The ROC curves illustrate the diagnostic ability of the microbial signatures, baseline HbA1c and C-peptide (areas under the ROC curves equal to 0.96, 0.71 and 0.66, respectively). HbA1c, glycohemoglobin; ROC, receiver-operating characteristic.

To address the gut microbiota and glycemic control in T2D patients, the 65 ASVs with differential abundance across subjects were significantly correlated with their HbA1c reduction ratios. After applying the random forest algorithm, 17 distinct features were selected (**Figures 3A, B**). The 6 updated scientific names that were adopted for 7 of the distinct features were: *Bacteroides dorei* (accession number: NR_041351.1), *Roseburia inulinivorans* (accession number: NR_042007.1), *Dialister succinatiphilus* (accession number: NR_041666.1), *Prevotella copri* DSM 18205 (accession number: NR_113411.1), *Butyricimonas virosa* (accession number: NR_041691.1) and *Mitsuokella multacida* (accession number: NR_027596.1). Among the 17 distinct features, we identified 9 species, 6 genera, 1 family (*Ruminococcaceae*) and 1 order (Bacteroidales) of taxonomy levels. The 4 distinct features in positive correlation with HbA1c reduction were defined as positive microbial signatures, whereas the 13 distinct features in negative correlation were defined as negative microbial signatures in GLP-1 RA treatment of T2D patients. The area under the receiver-operating characteristic curve (AUROC) of this microbiota-based classification was 0.96 (**Figure 3C**). In addition, the discrimination of traditional predictors including baseline HbA1c and C-peptide concentrations were also evaluated (AUROC were 0.71 and 0.66, respectively). Therefore, the significance of the distinct 17 microbial signatures to indicate a GLP-1 RA glycemic responder or non-responder was demonstrated.

### Prediction Models of GLP-1 RA Glycemic Responses

A linear regression model of HbA1c reduction was built with the 17 microbial signatures (Model 1) (**Table 2**). Five ASVs (*Eubacterium coprostanoligenes* sp., *Dialister succinatiphilus*.1, *Mitsuokella* sp.2, *Ruminococcaceae* sp., and *Dialister succinatiphilus*.2) were excluded due to high degrees of collinearity. This model was further adjusted with important clinical variables including baseline HbA1c concentration, UACR, total cholesterol and C-peptide (Model 2). Triglyceride concentration, dulaglutide or liraglutide user and concurrent use of insulin were excluded due to high degrees of collinearity. Model 2 revealed that *Bacteroides dorei*, *Lachnoclostridium* sp., *Mitsuokella multacida* in gut microbiota and baseline of HbA1c in patients were significant indicators for reductions in HbA1c in T2D patients treated with GLP-1 RA.

**Table 2:** Prediction of HbA1c reductions in type 2 diabetic patients receiving GLP-1 RA.

	Model 1			Model 2		
	B	SE	*P* value	B	SE	*P* value
*Bacteroides dorei*	0.431	0.125	0.001**	0.397	0.119	0.002**
*Lachnoclostridium* sp.	0.870	0.228	<0.001***	0.647	0.202	0.003**
*Butyricoccus* sp.	1.037	0.456	0.028*	NA	NA	NA
*Prevotella copri* DSM 18205	-0.566	0.155	0.001**	-0.318	0.180	0.086
Bacteroidales sp.	-1.184	0.257	<0.001***	NA	NA	NA
*Mitsuokella multacida*	NA	NA	NA	-0.755	0.264	0.007**
C-peptide	NA	NA	NA	0.151	0.086	0.088
Baseline HbA1c	NA	NA	NA	0.540	0.099	<0.001***

Model 1: microbial signatures only (R square = 0.593); Model 2: adjustment with clinical variables (R square = 0.724). B, regression coefficient; SE, standard error; NA, not applicable; HbA1c, glycohemoglobin. *P < 0.05; **P < 0.01; ***P < 0.001.

**Figure 4:**
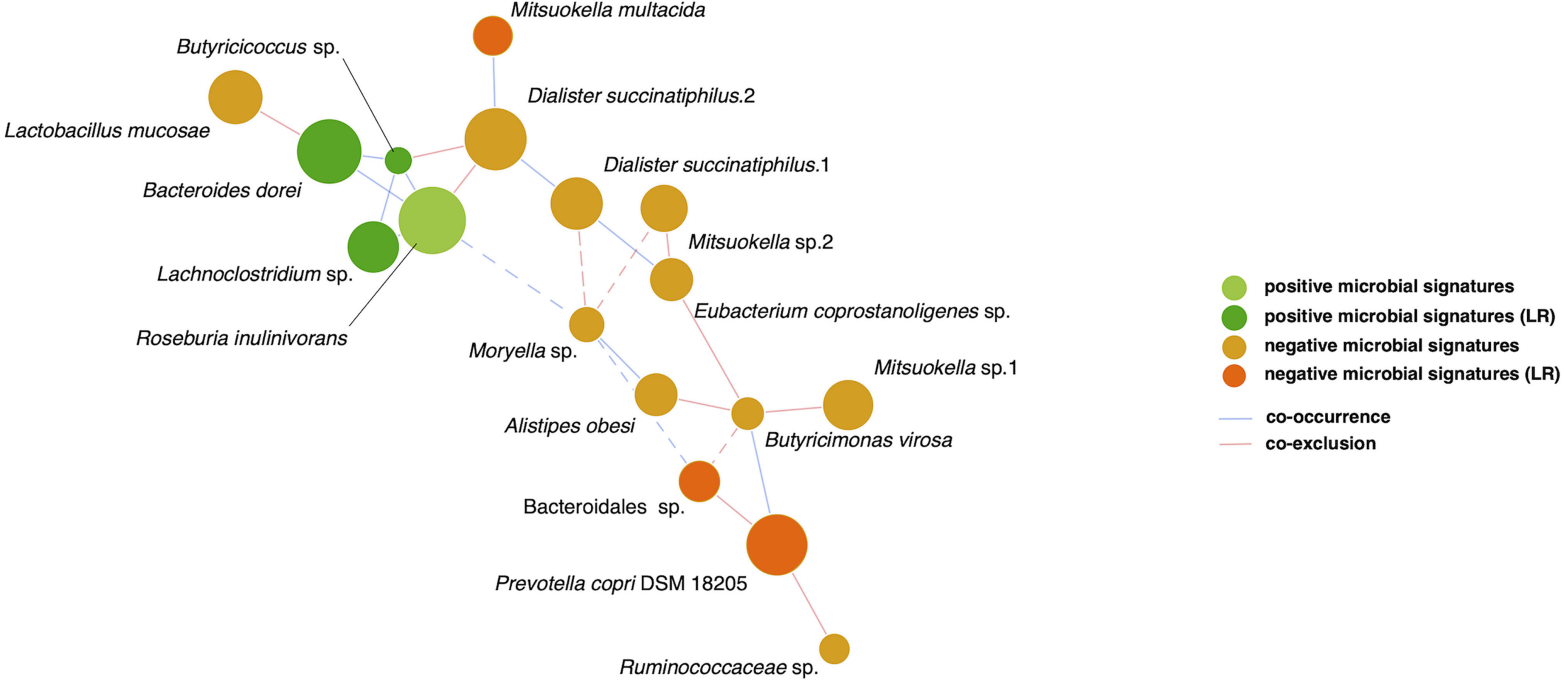
SparCC network of glycemic response-associated microbial signatures for GLP-1 RA treatment. The 17 distinct features with either positive correlation (green nodes) or negative correlation (orange nodes) to blood glucose reduction show co-occurrence (blue edges) or co-exclusion (red edges) between others in relationships. The 6 features remained in linear regression (LR) models are shown as deep color nodes. The size of a node is directly correlated to the respective abundance of microbial feature. The magnitude of the correlation is expressed as the inverse length of the respective edge. An absolute correlation magnitude < 0.2 (SparCC correlation) is presented in dotted edges.

Finally, relationships among the 17 microbial signatures were plotted in a network (**Figure 4**). Positive microbial signatures had close and positive interactions, whereas negative microbial signatures had relatively loose connections in small clusters. In conclusion, by adjusting for confounding effects of baseline HbA1c and β-cell function, *Bacteroides dorei* and *Lachnoclostridium* sp. served as representative positive signatures for GLP-1 RA glycemic responses, whereas *Mitsuokella multacida* served as a negative signature.

## Discussion

The gut microbiota was investigated in a cohort of T2D patients with poor glycemic control, features of diabesity, and at risk for cardiovascular diseases. Participants were regarded as a population that would benefit from GLP-1 RA therapy; nevertheless, glycemic responses to treatment were heterogeneous. Beta diversity in gut microbiota significantly differed between GLP-1 RA responders and non-responders. The top 17 microbial signatures correlated to HbA1c reductions in T2D patients treated with GLP-1 RA were identified and *Bacteroides dorei*, *Lachnoclostridium* sp., *Mitsuokella multacida* and *Prevotella copri* were all still remarkable after adjustment with clinical variables by linear regression model. This was apparently the first study characterizing impacts of gut microbiota composition on treatment efficacy of GLP-1 RA.

To analyze gut microbiota in our T2D cases with mild to moderate gut microbial dysbiosis, Bacteroidetes and Firmicutes were the dominant phyla, similar to healthy populations (41). The Firmicutes/Bacteroidetes ratio and alpha diversity were not associated with drug responsiveness in our T2D patients. These macro-metric tools often failed to differentiate T2D from healthy controls, perhaps due to the population heterogenicity of diabesity (35). The BMI of our cohort was overweight in both responders and non-responders. Nonetheless, microbial compositions had significantly different beta diversity, according to drug responsiveness. As a prominent genus within the Bacteroidetes phylum, *Bacteroides* has an important role in maintaining a healthy gut ecosystem (42) and in this study, relative abundance of *Bacteroides* was higher in GLP-1 RA responders. Conversely, a decrease of genera *Bacteroides* was a major observation associated with T2D (18). We inferred that a GLP-1 RA non-responder with lower relative *Bacteroides* abundance may indicate microbial dysbiosis.

*Bacteroides dorei* and *Roseburia inulinivorans*, the 2 identified positive microbial signatures at a species level, may cause immunomodulation (43, 44). *Bacteroides dorei* upregulated expression of tight junction genes in the colon and reduced microbial lipopolysaccharide production, thereby reducing gut permeability, and ameliorating endotoxemia (45). In contrast to healthy controls, there was a lower abundance of *Bacteroides dorei* in patients with coronary artery diseases (46). *Roseburia inulinivorans* had butyrate-producing and anti-inflammatory properties. Butyrate may modulate host energy expenditure by activating expression of peptide YY and GLP-1 in colonic epithelial cells (43). *Roseburia inulinivorans* has also been reported as a biomarker to discriminate healthy people from pulmonary tuberculosis patients and to predict remission of inflammatory bowel diseases (47, 48). In addition, *Bacteroides*, *Roseburia* and *Butyricicoccus* were reported as genera negatively associated with obesity and dyslipidemia (49). These findings were consistent with positive roles of *Bacteroides dorei*, *Roseburia inulinivorans* and *Butyricicoccus* sp. in GLP-1 RA treatment of T2D patients in our study.

The genus *Prevotella* is usually negatively correlated to genus *Bacteroides* within the Bacteroidetes phylum (42). *Prevotella copri* induces insulin resistance by augmenting circulating concentrations of branched-chain amino acids (50). The abundance of *Prevotella copri* was positively correlated to blood concentrations of interferon gamma and lipopolysaccharide in T2D patients (51). Interestingly, *Mitsuokella* was reported in association with dental infections (52). In a recent large cross-sectional analysis, both genera *Prevotella* and *Mitsuokella* were reported as trimethylamine-producing bacteria taxa (53). Trimethylamine N-oxide is closely linked to cardiometabolic diseases, including atherosclerosis and T2D (54). Therefore, *Prevotella copri* and *Mitsuokella* may be related to pathogenesis of T2D and its complications.

Some of the negative microbial signatures for GLP-1 RA treatment in T2D patients might be related to diseases. Four of six species of the *Dialister* genus were isolated from oral cavities, nasopharyngeal secretions and clinical samples and associated with dental infections or gastric carcinogenesis (55). *Dialister succinatiphilusis* was the first species isolated from human feces, but the clinical significance remains unclear (56). The *Alistipes* genus is isolated primarily from clinical samples and may be relevant to inflammation and cancer (57). *Alistipes obesi* was isolated from the fecal microbiota of an obese French patient (58). *Butyricimonas virosa* has caused bacteremia (59), the *Moryella* genus may be a biomarker for cervical intraepithelial lesions (60), and *Dialister*, *Alistipes*, *Butyricimonas virosa* and *Moryella* shared common features of pro-inflammatory or neoplastic diseases.

The increased relative abundance of *Bacteroides dorei* and *Roseburia inulinivorans* may indicate a lower inflammatory status of GLP-1 RA responders. Fermentation of indigestible polysaccharides by *Roseburia* may augment endogenous GLP-1 secretions that promote an incretin effect, and responders might harbor a favorable environment for GLP-1 action. Conversely, *Prevotella copri*, *Butyricimonas virosa*, *Mitsuokella*, *Dialister*, *Alistipes* or *Moryella* may indicate a profound inflammatory status of non-responders. The microbiota composition directly reflects human host health and determines therapeutic efficiency. GLP-1 RA modulates enteric immune responses as well as gut microbiota in murine models (61). Although humans have more heterogeneous microbial biodiversity, we inferred that the beneficial effects of GLP-1 action may have been obscured in non-responders due to profound dysbiosis and GLP-1 resistance.

In this study, microbial signatures had potential to serve as biomarkers for GLP-1 RA treatment response. However, there were some important limitations: this pilot study was cross-sectional with a limited scale and clinical confounders were not perfectly controlled. The early prescription of GLP-1 RA was restricted by regulations of National Health Insurance in Taiwan, probably due to the high medical cost. Thus, a small number of cases were enrolled in this study. Mean serum triglyceride concentration of responders was affected by outliers in thousand-level. Users of dulaglutide and liraglutide were analyzed together because the two agents had similarity in clinical indication and efficacy, and both were available for participants to choose. Nonetheless, clinical characteristics and beta diversity were different between the two subgroups (**Supplementary Table 1**, **Supplementary Figure 2**). Dulaglutide users were mostly injection-naïve, whereas nearly all liraglutide users had used insulin therapy before initiation of GLP-1 RA. Autoinjector pen or weekly injection might be more preferred for an injection-naïve patient during a process of shared decision making. Dulaglutide users had less severity in T2D, better glycemic responses, and lower abundances in some negative microbial signatures (**Supplementary Figure 3**). In addition, paired stool samples were collected in another cohort study to investigate the associations of GLP-1 RA effects and change of microbiota composition. Preliminary analysis detected no difference in beta diversity between baseline versus post-treatment (**Supplementary Figure 4**) and distinct microbial features between two drug users (**Supplementary Figure 5**), but more data are needed for further interpretation. Because metformin is known for modulating effects on microbiota, an analysis with exclusion of non-metformin users was performed. Most of the distinct microbial features could still be identified in top 17 rankings (**Supplementary Figure 6**). Besides, 16S amplicon rRNA sequencing for bacterial identification has detection limitations at species or strain levels and some key microbial features remained unclassified. Furthermore, gut microbiota may differ across ethnicity and habitat, making our results unreliable in every population. Nonetheless, gut microbial signatures for functional phenotyping in T2D patients has merit. Further prospective trials with shot-gun sequencing should be done.

In summary, gut microbiota was closely related to pathophysiology of T2D and GLP-1 resistance in this pilot study. Microbial compositions of T2D patients were heterogeneous among individuals. The HbA1c reduction following GLP-1 RA treatment was related to the gradient of the gut microbial dysbiosis, as confirmed in our study. The positive microbial signatures with immunomodulation effects were dominant in responders, whereas negative microbial signatures with pro-inflammatory properties were dominant in non-responders. Gut microbial signatures may not only predict GLP-1 RA efficacy but also reflect severity of T2D. Therefore, T2D management should emphasize promotion and maintenance of gut health.

## Data Availability Statement

The data sets presented in this study can be found in online repositories. The names of the repository/repositories and accession number(s) can be found below: https://www.ncbi.nlm.nih.gov/, PRJNA778609.

## Ethics Statement

The studies involving human participants were reviewed and approved by the institutional review board of Chang Gung Memorial Hospital. The patients/participants provided their written informed consent to participate in this study.

## Author Contributions

C-YT, C-HL, and C-NT designed the study. C-YT and C-HL enrolled participants. C-YT and Y-HC collected data. C-YT and H-CL processed/researched the data and wrote the manuscript. Y-HC performed DNA extraction. P-YL assisted data research. H-YC organized food frequency questionnaire interviews. M-CH designed the food frequency questionnaire and conducted nutrients transformation. Y-HC, P-YL, H-YC, M-CH, C-HL, and C-NT contributed to discussion. C-HL and C-N.T supervised the study and reviewed/edited the manuscript. All authors contributed to the article and approved the submitted version.

## Funding

This work was supported by grants from the Chang Gung Memorial Hospital (CMRPG3J1021, CMRPG3K1171) awarded to C-YT.

## Conflict of Interest

The authors declare that the research was conducted in the absence of any commercial or financial relationships that could be construed as a potential conflict of interest.

## Publisher’s Note

All claims expressed in this article are solely those of the authors and do not necessarily represent those of their affiliated organizations, or those of the publisher, the editors and the reviewers. Any product that may be evaluated in this article, or claim that may be made by its manufacturer, is not guaranteed or endorsed by the publisher.
