# Supplementary material for: Gut Microbial Signatures for Glycemic Responses of GLP-1 Receptor Agonists in Type 2 Diabetic Patients: A Pilot Study
Source: Front Endocrinol (Lausanne). 2022 Jan 10;12:814770. doi: 10.3389/fendo.2021.814770 (PMC8793908; doi:10.3389/fendo.2021.814770)
Supplement: Supplementary file 1 [file DataSheet_1.pdf]

Supplementary Material

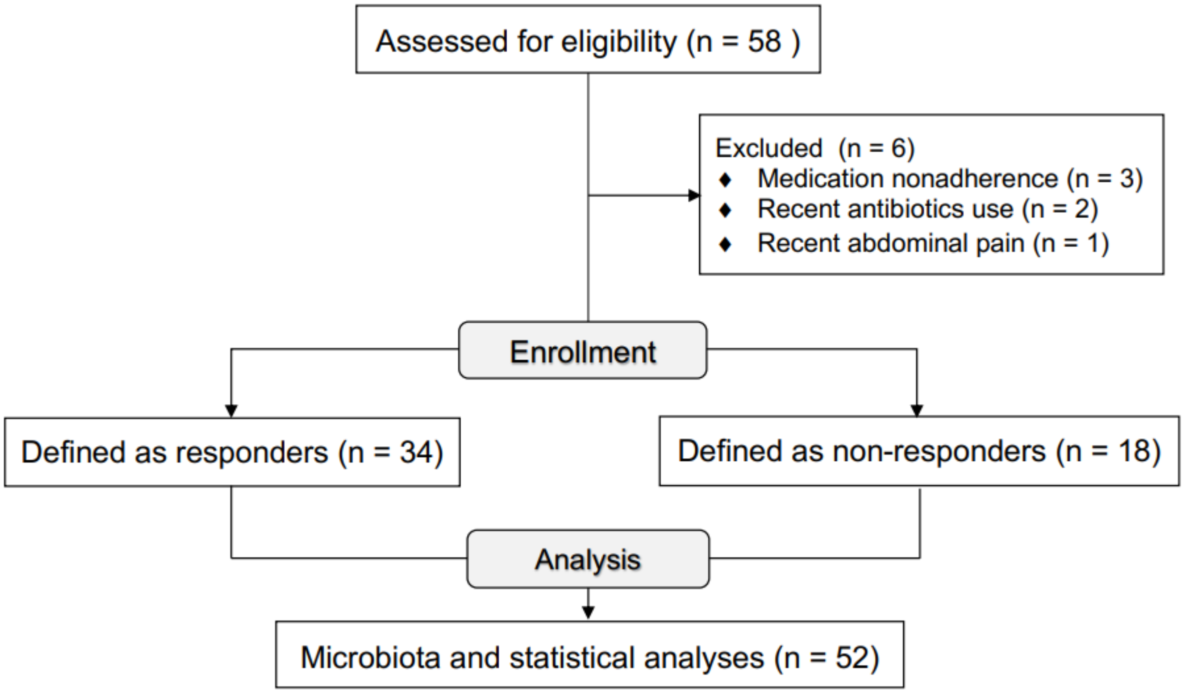

Supplementary Figure 1. Flow diagram of participant enrollment.

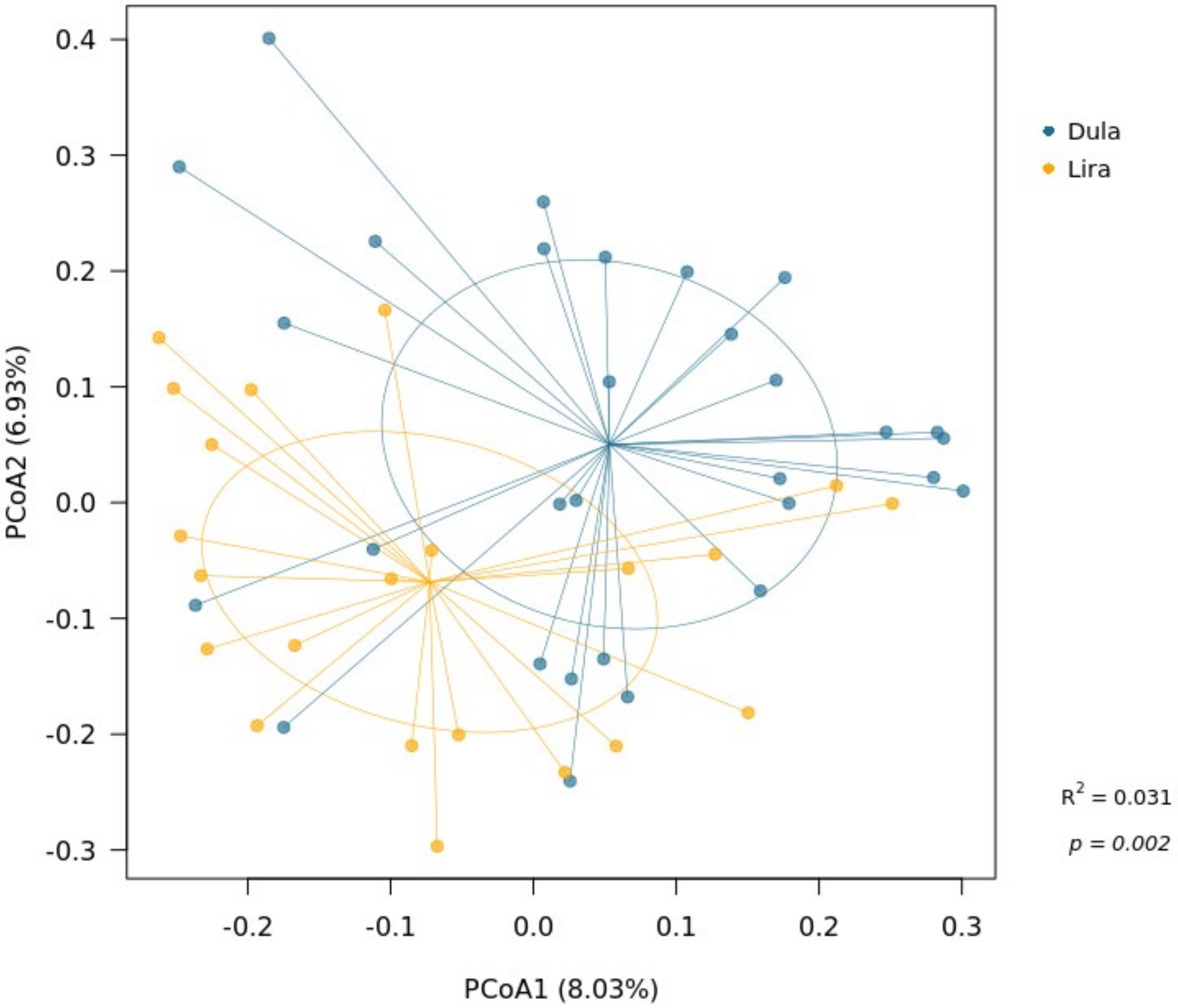

Supplementary Figure 2. Microbiota biodiversity of type 2 diabetic patients with different GLP-1 RA. There was a difference in beta diversity between dulaglutide users (Dula, n = 30) versus liraglutide users (Lira, n = 22) by principle coordinate analysis (*P* = 0.002, ADONIS). Each point represented 1 individual, colored according to drug name.

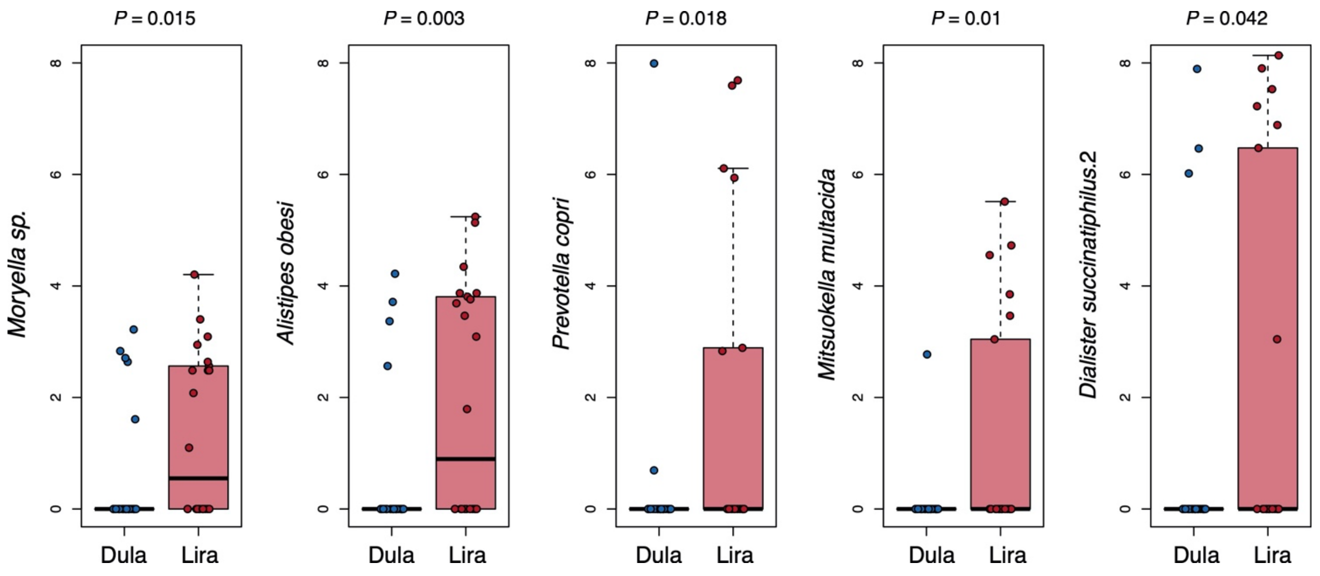

Supplementary Figure 3. The log-transformed abundances of microbial features in different GLP-1 RA users: dulaglutide users (Dula, n = 30) versus liraglutide users (Lira, n = 22). All of the 17 distinct features were compared. Only the features that had *P* values < 0.05 are shown (Wilcoxon rank sum test).

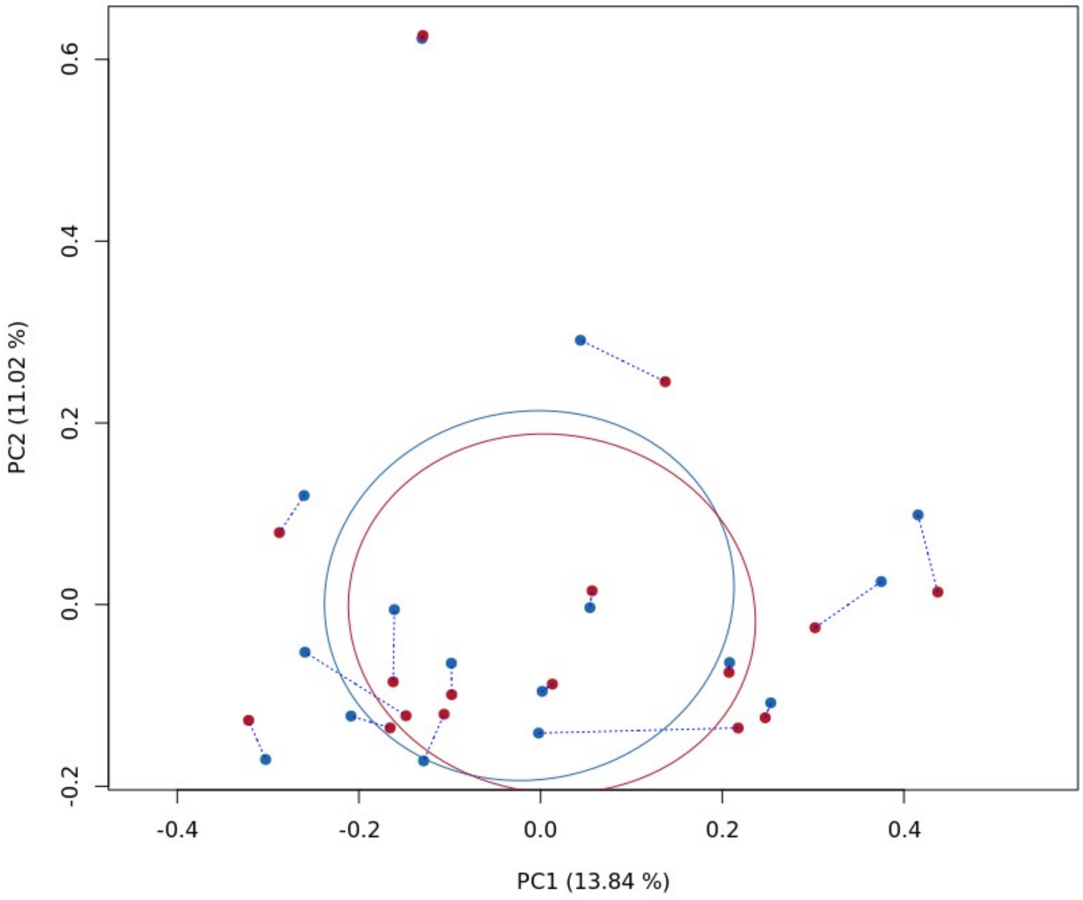

Supplementary Figure 4. Microbiota biodiversity of type 2 diabetic patients in baseline and post-GLP-1 RA treatment. Baseline stool samples from 16 of 52 participants (8 dulaglutide and 8 liraglutide users) were collected in another cohort study and preliminary analysis detected no difference in beta diversity between baseline versus post-treatment by principle coordinate analysis (*P* = 0.63, ADONIS). Each point represented each sampling of individuals, colored according to sampling time. Groups are baseline (blue) and post-treatment (red). Lines connect specimens of 1 individual. The circles of each cluster are closely overlapping.

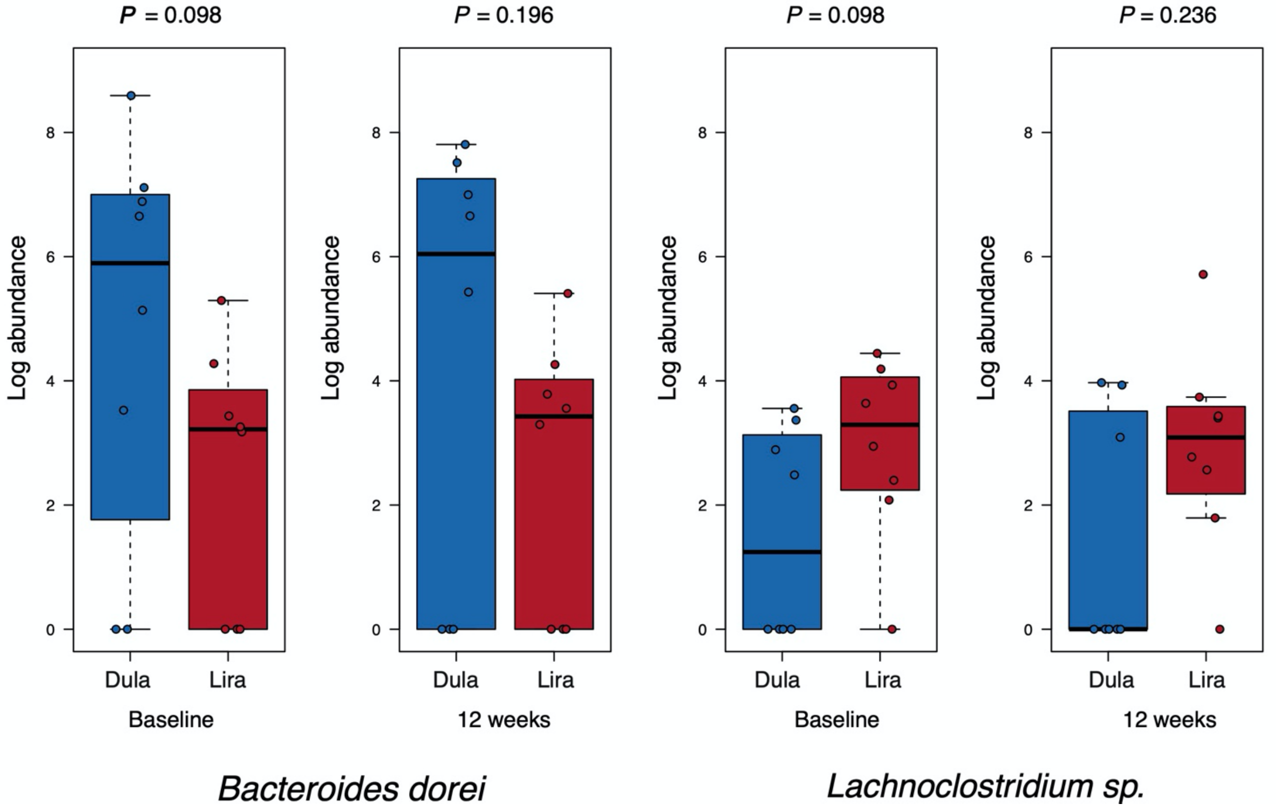

Supplementary Figure 5. The log-transformed abundances of microbial features at baseline and 12-week treatment: dulaglutide users (Dula, $n = 8$) versus liraglutide users (Lira, $n = 8$). All of the 17 distinct features were compared. Only the features that had P values < 0.1 are shown (Wilcoxon rank sum test). There were 6 and 7 responders in dulaglutide users and liraglutide users, respectively.

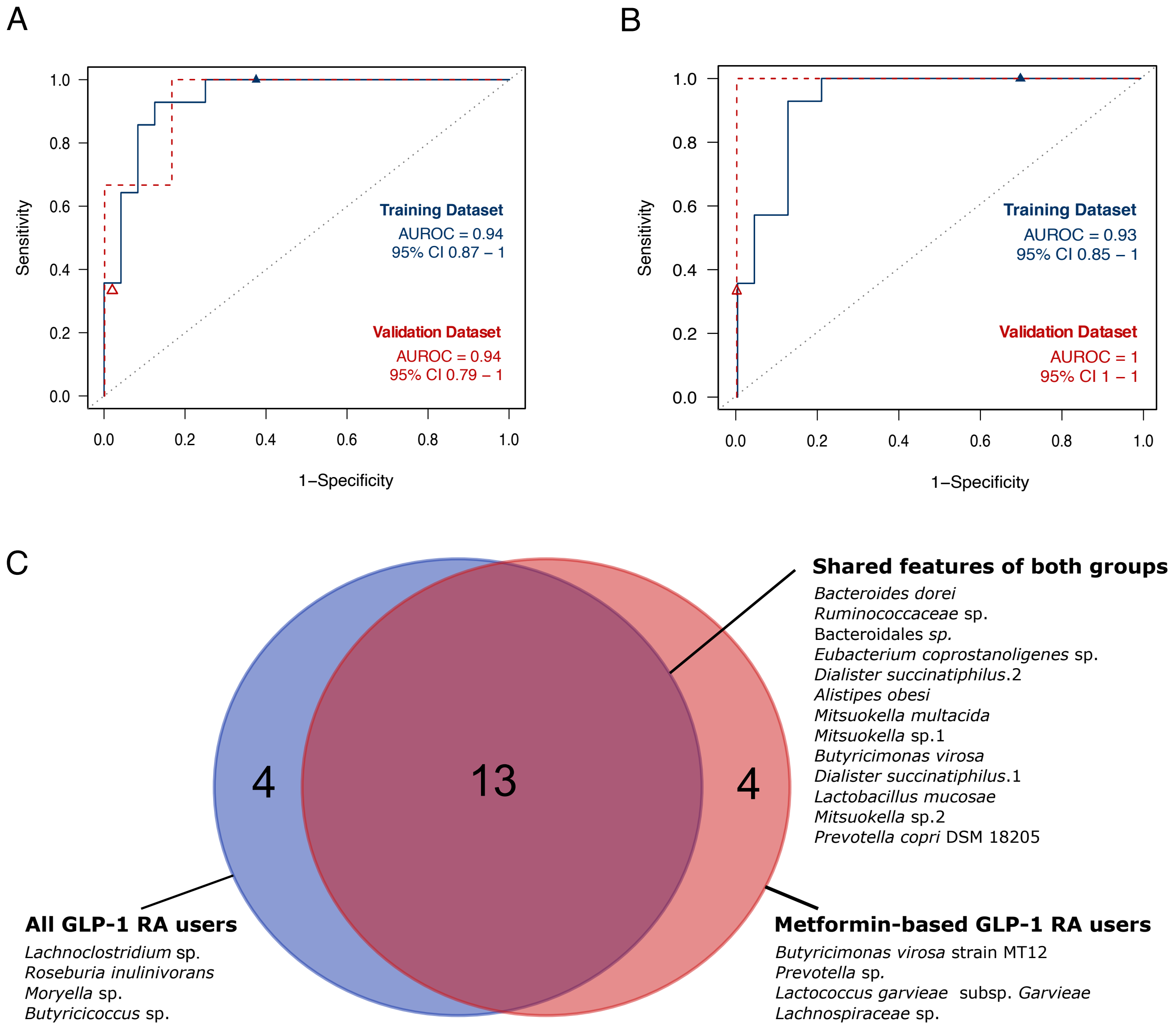

Supplementary Figure 6. Validation of different microbial signatures for metformin-based GLP-1 RA users (n = 47). (A) The ROC curves illustrate the diagnostic ability of the top 17 microbial signatures that were identified in metformin-based GLP-1 RA users. (B) The ROC curves illustrate the diagnostic ability of the original 17 microbial signatures that were derived from all GLP-1 RA users. (C) The Venn diagram shows the common and unique features in the two types of microbial signatures (blue circle: all GLP-1 RA users, red circle: metformin-based GLP-1 RA users). ROC, receiver-operating characteristic. AUROC, the area under the ROC curve.
