# Supplementary material for: Gut Microbial Signatures for Glycemic Responses of GLP-1 Receptor Agonists in Type 2 Diabetic Patients: A Pilot Study
Source: Front Endocrinol (Lausanne). 2022 Jan 10;12:814770. doi: 10.3389/fendo.2021.814770 (PMC8793908; doi:10.3389/fendo.2021.814770)
Supplement: Supplementary file 2 [file Table_1.docx]

Supplementary Material

**Supplementary Table 1. Clinical characteristics of type 2 diabetic patients with different GLP-1 RA (baseline).**

|  | Dulaglutide  (n = 30) | Liraglutide  (n = 22) | *P* value |
| --- | --- | --- | --- |
| Age (years) | 52.4 ± 1.9 | 53.6 ± 3.0 | 0.985 |
| Gender (male/female) | 18/12 | 12/10 | 0.780 |
| Duration of diabetes (years) | 9.8 ± 1.0 | 11.8 ± 1.3 | 0.294 |
| BMI | 30.4 ± 0.9 | 30.2 ± 1.1 | 0.788 |
| Fasting plasma glucose (mg/dL) | 189.9 ± 12.0 | 158.0 ± 12.6 | 0.049* |
| HbA1c (%) | 9.4 ± 0.3 | 9.8 ± 0.4 | 0.585 |
| eGFR (mL/min/1.73 m²) | 78.7 ± 7.8 | 97.7 ± 9.5 | 0.239 |
| U-ACR | 921 ± 421 | 444 ± 253 | 0.824 |
| ALT (U/L) | 45.0 ± 6.2 | 31.1 ± 4.9 | 0.116 |
| Total cholesterol (mg/dL) | 188.6 ± 12.6 | 170.0 ± 9.8 | 0.594 |
| LDL-cholesterol (mg/dL) | 96.3 ± 7.7 | 98.0 ± 7.4 | 0.676 |
| HDL-cholesterol (mg/dL) | 37.9 ± 2.1 | 41.7 ± 2.3 | 0.266 |
| Triglyceride (mg/dL) | 346.5 ± 63.8 | 197.5 ± 62.2 | < 0.001** |
| C-peptide (ng/mL) | 4.0 ± 0.4 | 2.0 ± 0.3 | < 0.001** |
| Estimated calorie intake (Kcal/d) | 1630 ± 117 | 1629 ± 120 | 0.707 |
| Protein (%) | 17.3 ± 0.8 | 17.7 ± 0.7 | 0.402 |
| Fat (%) | 32.6 ± 1.8 | 31.7 ± 1.9 | 0.795 |
| Carbohydrate (%) | 50.1 ± 2.5 | 50.7 ± 2.4 | 0.941 |
| Responder/non-responder | 23/7 | 11/11 | 0.076 |
| Concurrent medication (use/no-use): | |  |  |
| Metformin | 28/2 | 19/3 | 0.639 |
| Sulphonylurea | 16/14 | 4/18 | 0.020* |
| Pioglitazone | 5/25 | 1/21 | 0.226 |
| Insulin | 3/27 | 20/2 | < 0.001** |

BMI, body mass index; HbA1c, glycohemoglobin; eGFR, estimated glomerular filtration rate; UACR, urine albumin-to-creatinine ratio; ALT, alanine transaminase; LDL, low-density lipoprotein; HDL, high-density lipoprotein. Continuous Data are shown as means ± standard error of the mean. Wilcoxon rank sum test / Fisher’s Exact Test, **P* < 0.05; ***P* < 0.01.
